# Electricity in Uterine Displacements*Read before the Albany Academy of Medicine.

**Published:** 1883-08

**Authors:** E. A. Bartlett

**Affiliations:** Albany


ELECTRICITY IN UTERINE DISPLACEMENTS.* By E. A. BARTLETT, M. D., Albany.

The frequency with which disease of the uterus and its appendages are prominent causes in the ill health of women is erroneously regarded by physicians. There are those who see these as causes in every ailing woman, and there are others who seem never to take them into consideration Others, from feelings of delicacy in approaching the subject, or for other reasons, try to make themselves believe that there is some other cause for the obscure symptoms by which they are confronted.

These are all false positions. However rarely or frequently they may be factors in ill health, it is the experience of every practitioner that uterine diseases may be the source of many remote, subjective symptoms ; and, if a patient has persistent mysterious symptoms for which no adequate cause can be found, and which do not give way under a general course of treatment, we are not only justified in directing our attention to the pelvis, but we are in duty bound to do so. In many cases the pain in the loins, dragging of the viscera, almost constant backache and other characteristics tell too plainly where the seat of trouble is. Mal-positions are found in a large percentage of these cases, and it is to the *rationale* of electricity in their treatment that your attention is invited.

Adopting Simpson's division, we have two classes of displacements—*versions* and *flexions*, the latter being not an exaggeration of the former, but an entirely separate condition. In the former the uterus may be turned upside down and still the canal be relatively straight, while in the latter the canal is bent upon

*Read before the Albany Academy of Medicine.

itself at more or less of an angle. This fact is important, because it points to various causations, and necessitates treatment peculiar to the case. These mal-positions may be due to a variety of causes. In the poorly developed uterus anteversion is about in proportion to the want of development, but in far the greater number of cases they are due to congestion, engorgement and hypertrophy. The veins of the uterus are normally of large size, and as a result of carelessness during the puerperal and menstrual periods they are only partially emptied, venous congestion takes place, the organ is engorged, and there may follow hypertrophy of tissue. Any cause producing relaxation of the suspensory ligaments and the vaginal walls finds an admirable ally in this overburdened uterus, and version is the result. If, however, there has taken place degeneration in the muscular fiber of the uterus, flexion will be the result.

It cannot be denied that the general treatment by a prolonged course of chalybeates and stimulation of the uterus, while successful in some cases of displacement from a partially developed uterus, is in more of the same kind totally inadequate. It is only too true that in the hands of very many practitioners the use of pessaries, whose number and form is legion, is followed by the most unsatisfactory and sometimes the most unhappy results. It is the experience of the very best gynæcologists that elytrorrhaphy, scarification and such like operations are insufficient in many cases to bring about desired results. And so it happens that, without thought of superseding any of these methods mentioned, electricity has been enrolled in the list of useful remedies. For a long time it had been used by persons outside the medical profession, and indeed it was the beneficent results so obtained that centered the professional mind upon its use in this special department. Much of this use has been empirical; the details of its action were not sought for; the excellent results sufficed.

We know that electricity acts, when passed through the human body, either mechanically or chemically, either directly upon the organs or indirectly through the nerves. Now, if we remember what conditions exist in a displacement and some of the principal causes, we shall surmise that the use of electricity will be followed by very satisfactory results in many cases, but we shall also realize that there will occur many cases in which electricity alone would be just as powerless as any other application. In an extreme case of version or flexion it is manifest any conceivable application of electricity would, of itself, be useless to accomplish reposition. Were the supports to the uterus composed of muscular fiber, and were they in a condition of extreme relaxation, there might be a possibility of causing sufficient contraction to bring the organ into place. This is not the case; a portion of the ligaments supporting the uterus are simply folds of peritoneum containing no muscular fiber of their own, while others are the usual ligamentous tissue; both have some muscular fiber prolonged upon them from the uterine walls, which may be excited sufficiently to render some service in slight versions, but in severe or extreme cases they are of no appreciable service.

The foregoing is a true statement of facts; nevertheless, experience teaches us that an ante-, retro- or lateroverted uterus, if not too far gone, can be replaced under the use of electricity, and this, in many cases, applied only exteriorly. How is this result produced? Placing one electrode of a faradic machine upon the lower lumber vertebræ and the other upon either inguinal or hypogastric region, let the circuit through the primary coil be closed; there will be felt, if the current be sufficiently strong, a sharp contraction under the latter electrode. This has by some been assumed to be the action of the uterus and its supports; the assumption, however, is manifestly erroneous. As we have seen there are no muscular fibers in the uterine supports to contract, or at least not enough to cause so violent a contraction; and the uterus lies so deep in the pelvis that the contraction of its fibers would be much less perceptible externally than what we notice. The assumption is partly due to the results obtained in the obstetric use of electricity, where the contractile effects upon the uterine walls are of such marked character. The eases are not parallel, for during parturition the uterus is in the abdominal cavity, where its contractions can be plainly felt, which is not the case here.

If we look at the anatomical disposition of the parts, we shall find that we have under the electrode, when it is in the left inguinal region, the superficial and deep abdominal muscles and below them the sigmoid flexure of the colon; in the right inguinal region the corresponding muscles and beneath, the cæcum; in the hypogastric region, the rectus abdominis and pyramidalis muscles and beneath, the bladder. If we place this electrode upon any portion of the abdomen we shall note, under the same conditions, the same phenomena of contraction, and, if the action be prolonged and extended over the whole abdomen, it will bring about the act of defecation, or a desire for it. This would tend to show that the contraction here takes place in the muscles which constitute the abdominal parietes and also in the muscular coat of the intestines. What is true of this part is also true of the other part of the abdomen. The contraction felt there is not in the uterus and its supports. If, however, we insert a finger in the vagina and place it in contact with the uterus, we shall feel a slight thrill and decided contraction of the walls of the uterus analagous to the sensation received during a labor pain; but this contraction is not sufficient nor in the proper tissue to replace the organ, supposing it to be displaced; so we must seek further for the remedial cause.

Examples are not wanting of the power of electricity, both galvanic and faradic, to relieve engorged and even hypertrophied tissue. Galvanism, by its subtle chemical, and faradization by its mechanical action, are capable of improving nutrition, stimulating muscular contraction and hastening absorption. Here, then, we have a rational and tenable method by which electricity may be of great service. The modification of nutrition caused by electricity may have two opposite effects; it may cause increase or it may caused iminution in the size of a part or organ. In these opposite results there is nothing inconsistent; they are the result of healthy nutrition caused by the current.

If we place the electrodes as before, the hypogastric plexus of nerves, from which pass the nerves innervating the uterus and vagina, lying directly in front of the sacrum, is in the field of action for the current. It is just here that Budge and Waller located the genito-spinal center. The electricity conducted along these nerves to the uterus and vagina there does its remedial work, contracting muscles, altering nutrition and increasing the tone of the relaxed ligaments. Thus stimulated, the displaced organ regains its normal size, and, slowly perhaps, but surely, returns to its proper place.

This method of external application is especially serviceable in vaginal cases and with timid women, but in very rebellious cases, and with married women generally, the introduction of an electrode to the vagina will be found much more efficacious. Not only is the muscular coat of the vaginal walls made to recover its tone, and thus one of the best supports provided for the uterus, but an internal application brings the organs directly under the action of such pole as their condition may require, and enables us to produce somewhat more rapidly tissue changes. Moreover, the chemical effects of the galvanic current upon the secretions of the mucous membrane and the contiguous glands may be utilized by this means, remembering the differential polar action. In like manner, the insertion of one electrode into the rectum or bladder enables the operator to localize the action very strictly and produce marked results.

It is well known that if a strong galvanic current, passing through the pelvic viscera, be suddenly interrupted there will be produced a "coppery" taste in the mouth. This phenomenon may be made to "point a moral." The hypogastric plexus, of which we have spoken, recieves fibers from the spinal nerve, and the vaginal branch has a large number. The diffusion of galvanism along these fibers to the nerves, and thus to the cord, serves to put the spinal cord in an electronic condition, and this irritability modifies decidedly the subjective symptoms accompanying uterine disease. This is really an important feature in the *rationale* of electricity in uterine displacements, and accounts somewhat for the prompt relief which so often follows this kind of treatment, of the despondency accompanying these cases, and which is one of the great drawbacks to calling the attention of a young woman to the fact that she is a subject of "female weakness." But it would be unwise to depend upon this "diffusion of current" for relief of subjective and remote symptoms. The operator who confines himself to localized treatment of uterine disease, neglecting thorough general treatment, sacrifices much of the beneficial effect of electricity. If it is necessary to produce mechanical effects, local faradization is indicated ; for local chemical effects, galvanism ; but this is only a small part of the work of restoration. The connection with, and relation to, other organs and parts of the body of the organs of generation are so intimate, and derangement here is followed by so much disturbance elsewhere that general treatment of some sort becomes essential. In the form of electricity addressed either to the cerebro spinal axis or the sympathetic system we have a valuable auxiliary whose use hastens recovery. In conclusion, time is essential in any form of treatment for these cases, and the patient may be given to understand that relief is not the result of a few days, but of weeks or months.—*Med. Annals.*

